# Novel leukocyte-depleted platelet-rich plasma-based skin equivalent as an in vitro model of chronic wounds: a preliminary study

**DOI:** 10.1186/s12860-021-00366-6

**Published:** 2021-05-10

**Authors:** Elisa Seria, George Galea, Joseph Borg, Kevin Schembri, Gabriella Grech, Sarah Samut Tagliaferro, Alexander Felice

**Affiliations:** 1 Department of Physiology and Biochemistry and Centre of Molecular Medicine and Biobanking, Faculty of Medicine and Surgery, University of Malta, Msida, MSD2080 Malta; 2 National Blood Transfusion Centre and Department of Pathology, University of Malta, Msida, MSD2080 Malta; 3 Department of Applied Biomedical Science, Faculty of Health Sciences, University of Malta, Msida, MSD2080 Malta; 4 Department of Surgery, Faculty of Medicine and Surgery, University of Malta Medical School and Mater Dei Hospital, Msida, MSD2080 Malta

**Keywords:** Wound healing, Biomaterials, Platelet rich plasma, Skin equivalent, Inflammation

## Abstract

**Background:**

Chronic leg ulcerations are associated with Haemoglobin disorders, Type2 Diabetes Mellitus, and long-term venous insufficiency, where poor perfusion and altered metabolism develop into a chronic inflammation that impairs wound closure. Skin equivalent organotypic cultures can be engineered in vitro to study skin biology and wound closure by modelling the specific cellular components of the skin. This study aimed to develop a novel bioactive platelet-rich plasma (PRP) leukocyte depleted scaffold to facilitate the study of common clinical skin wounds in patients with poor chronic skin perfusion and low leukocyte infiltration. A scratch assay was performed on the skin model to mimic two skin wound conditions, an untreated condition and a condition treated with recombinant tumour necrotic factor (rTNF) to imitate the stimulation of an inflammatory state. Gene expression of *IL8* and *TGFA* was analysed in both conditions. Statistical analysis was done through ANOVA and paired student t-test. *P* < 0.05 was considered significant.

**Results:**

A skin model that consisted of a leukocyte-depleted, platelet-rich plasma scaffold was setup with embedded fibroblasts as dermal equivalents and seeded keratinocytes as multi-layered epidermis. Gene expression levels of *IL8* and *TGFA* were significantly different between the control and scratched conditions (*p* < 0.001 and *p* < 0.01 respectively), as well as between the control and treated conditions (p < 0.01 and *p* < 0.001 respectively). The scratch assay induced *IL8* upregulation after 3 h (*p* < 0.05) which continued to increase up to day 1 (*p* < 0.05). On the other hand, the administration of TNF led to the downregulation of *IL8* (*p* < 0.01), followed by an upregulation on day 2. *IL8* gene expression decreased in the scratched condition after day 1 as the natural healing process took place and was lower than in the treated condition on day 8 (*p* < 0.05).

Both untreated and treated conditions showed a downregulation of *TGFA* 3 h after scratch when compared with the control condition (*p* < 0.01). Administration of rTNF showed significant downregulation of *TGFA* after 24 h when compared with the control (*p* < 0.01) and treated conditions (*p* < 0.05).

**Conclusion:**

This study suggests that a leukocyte-depleted PRP-based skin equivalent can be a useful model for the in vitro study of chronic skin wounds related to poor skin perfusion.

**Supplementary Information:**

The online version contains supplementary material available at 10.1186/s12860-021-00366-6.

## Background

The incidence of frequent leg ulcers increases with age and is common in age, leading to a negative impact on the patient’s quality of life and a considerable cost for the health care service [1]. Haemolytic disorders such as Sickle Cell Disease and β-thalassaemia are seldomly diagnosed in Malta and other Mediterranean countries due to a vast occurrence of β globin gene mutations [2]. Haemoglobin disorders are associated with chronic cutaneous wounds due to peripheral hypoxia [3, 4], lower bioavailability of nitric oxide (NO), iron overload, and an impaired endothelial function [5]. Chronic leg ulceration has also been seen in Type 2 Diabetes Mellitus (T2DM) and in long-term venous insufficiency where poor perfusion and altered metabolism set up a chronic inflammation that impairs repair and wound closure [6, 7].

A skin model scaffold, based on a three-dimensional organotypic culture [8], can be used to study the process of wound healing such as for skin biology studies and testing of topically applied products. The epithelial cells and the fibroblasts of the skin equivalent are capable of secreting cytokines, chemokines, and growth factors that mimic wound healing and favour skin regeneration, to provide a protective layer over the wound [9, 10].

The objectives of this study were to generate a novel bioactive platelet-rich plasma (PRP)-leukocyte-depleted scaffold to develop an in vitro model of a typical clinical wound of patients with poor chronic skin perfusion and low leukocyte infiltration. An air-liquid interface model was used to reproduce a full-thickness skin model consisting of a co-culture of epithelial cells and fibroblasts, seeded into a PRP-leukocytes-depleted scaffold. The PRP leukocyte-depleted scaffold was stimulated with calcium chloride (CaCl2), which acted as a platelet activator, triggering the establishment of autologous thrombin from prothrombin. This facilitated the formation of a fibrin matrix that provided the support structure [11], as well as a source of growth factors, including platelet-derived growth factor (PDGF), transforming growth factor-β (TGFB), epidermal growth factor (EGF) and vascular endothelial factor (VEGF). These growth factors are involved in haemostasis, wound healing and tissue regeneration in various injured tissues [12]. The scaffold was leukocyte depleted to avoid the unnecessary and excessive cytokine expression from white blood cells, that may delay or impede the wound healing process. Although an inflammatory response is fundamental for wound healing, a subset of inflammatory cells may result in delayed healing [13]. Furthermore, leukocyte-depleted PRP is more predictable, as growth factor release and fibrin scaffold integrity are conserved when exposed to inflammatory conditions [14].

Recombinant TNF (rTNF) was administered to the treated wounded skin model in order to mimic an inflammatory condition. TNF is a cytokine with pleiotropic effects on cell growth, inflammation, and immune responsiveness. The local effects of TNF are usually beneficial for the host, but when generated at higher concentrations within chronic inflammatory lesions, the proinflammatory effects of TNF often become deleterious and systemic [15].

The expression of the genes encoding for IL-8 and transforming growth factor-α (TGFA), which are involved in the inflammatory phase [16] and the proliferative phase [17] of wound healing, were analysed to evaluate how the leukocyte depleted PRP-based skin equivalent, with or without the rTNF administration can affect the final therapeutic outcomes in an inflammatory state.

## Results

### Successful isolation and culture of primary epithelial cells, fibroblasts, and preparation of the skin equivalent

The skin equivalent model was assembled through a co-culture of epithelial cells and fibroblasts seeded into the PRP-leukocyte depleted scaffold and cultured in an air-liquid interface system, stimulating the development of a multi-layered skin equivalent.

**Fig. 1:**
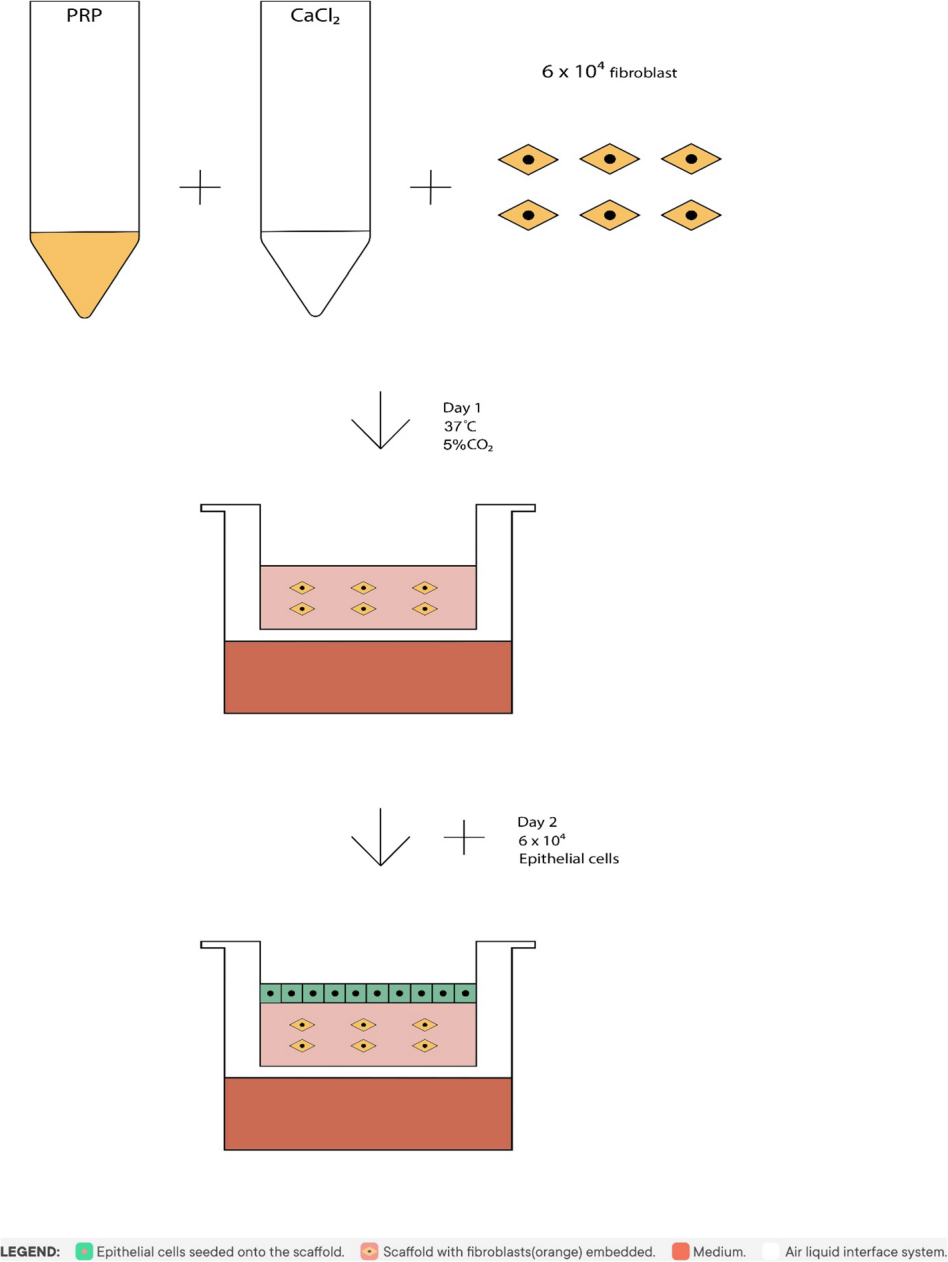
Preparation of the leukocyte-depleted PRP-based skin equivalent. The platelet rich plasma (PRP) was mixed with 20 mM calcium chloride (CaCl2) solution and 6 × 104 cells/cm2 fibroblasts. The mixed solution was poured into a 24 mm trans-well at 37 °C in a CO2 incubator for 24 h. The CaCl2 worked as activator for the formation of autologous thrombin and allowed the PRP-based derma equivalent to solidify. At day 2 the epithelial cells were seeded on the PRP-based derma equivalent with a density of 6 × 104 cells/cm2. This skin model was cultured with an air-liquid interface system allowing the development of a multi-layered skin equivalent

A representative scheme is illustrated in Fig. 1.

**Fig. 2:**
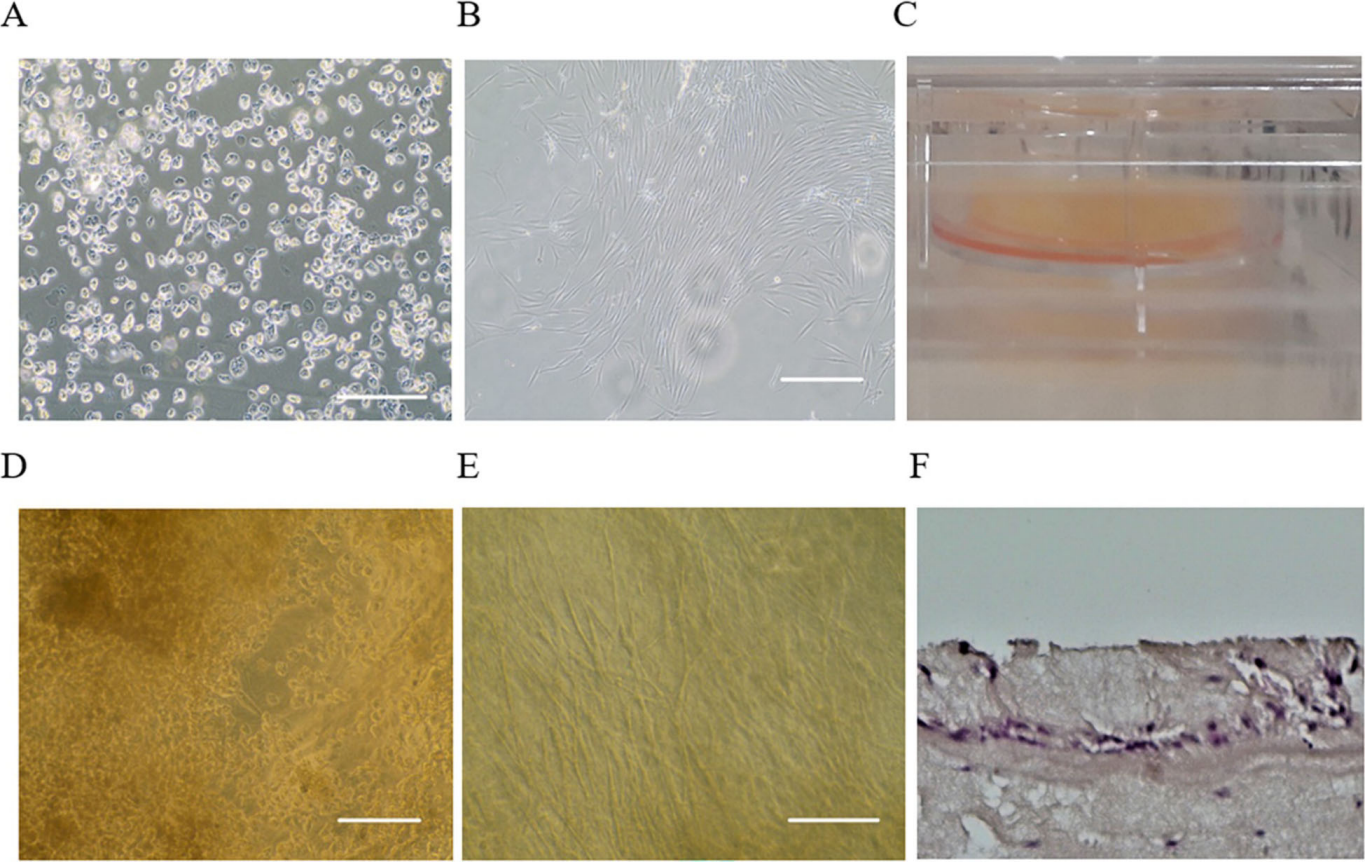
Morphology of the primary epithelial cells, fibroblasts, and skin equivalent. **a** Seeded epithelial cells showing their characteristic “pavement stone” morphology; **b** Fibroblasts reaching confluence 15 days after the plating of the dermal cells; **c** Typical air-liquid interface system composed by a PRP-leukocyte depleted scaffold in a 24 mm trans-well. The skin model was assembled through a co-culture of epithelial cells and fibroblasts-seeded in the PRP-leukocyte depleted scaffold; **d** Confluent epithelial cells growing in multilayers on the surface of the scaffold; **e** Confluent fibroblasts embedded into the scaffold; **f** Transversal section of the skin equivalent showing the presence of different epithelial cells organized into the different layers of the skin. Nuclei of the epithelial cells are stained in violet. The image also shows the presence of the fibroblasts (nuclei stained in violet) embedded in the PRP-leukocyte depleted scaffold stained in pink reassembling the extracellular matrix of the skin. Scale bar = 100 μm

The isolated epithelial cells displayed a short and wide spindle-shape and epithelial morphology similar to a “pavement stone” (Fig. 2a). The cultures reached confluence and were ready to passage within 2 weeks. The growth of fibroblasts from the dermal tissue was observed by day 10 after tissue plating. The dermal tissue was removed from the plate 15 days after plating, where the proliferating fibroblasts initiated the primary culture reaching confluence at day 20 after plating (Fig. 2b).

Figure 2c shows the assembled skin equivalent model cultured in an air liquid interface system. Microscopical observation of the scaffold confirmed the presence of confluent and multi-layered epithelial cells (Fig. 2d), and the presence of proliferating spindle-shaped fibroblasts (Fig. 2e) on the PRP scaffold. Histological examination of the skin equivalent showed the presence of different epithelial cells organized in a typical skin tissue. The nuclei of the epithelial cells and the fibroblasts were identified using Mayer’s Haematoxylin-Eosin stain (Fig. 2f).

### Characterization of primary culture of epithelial cells and fibroblasts

**Fig. 3:**
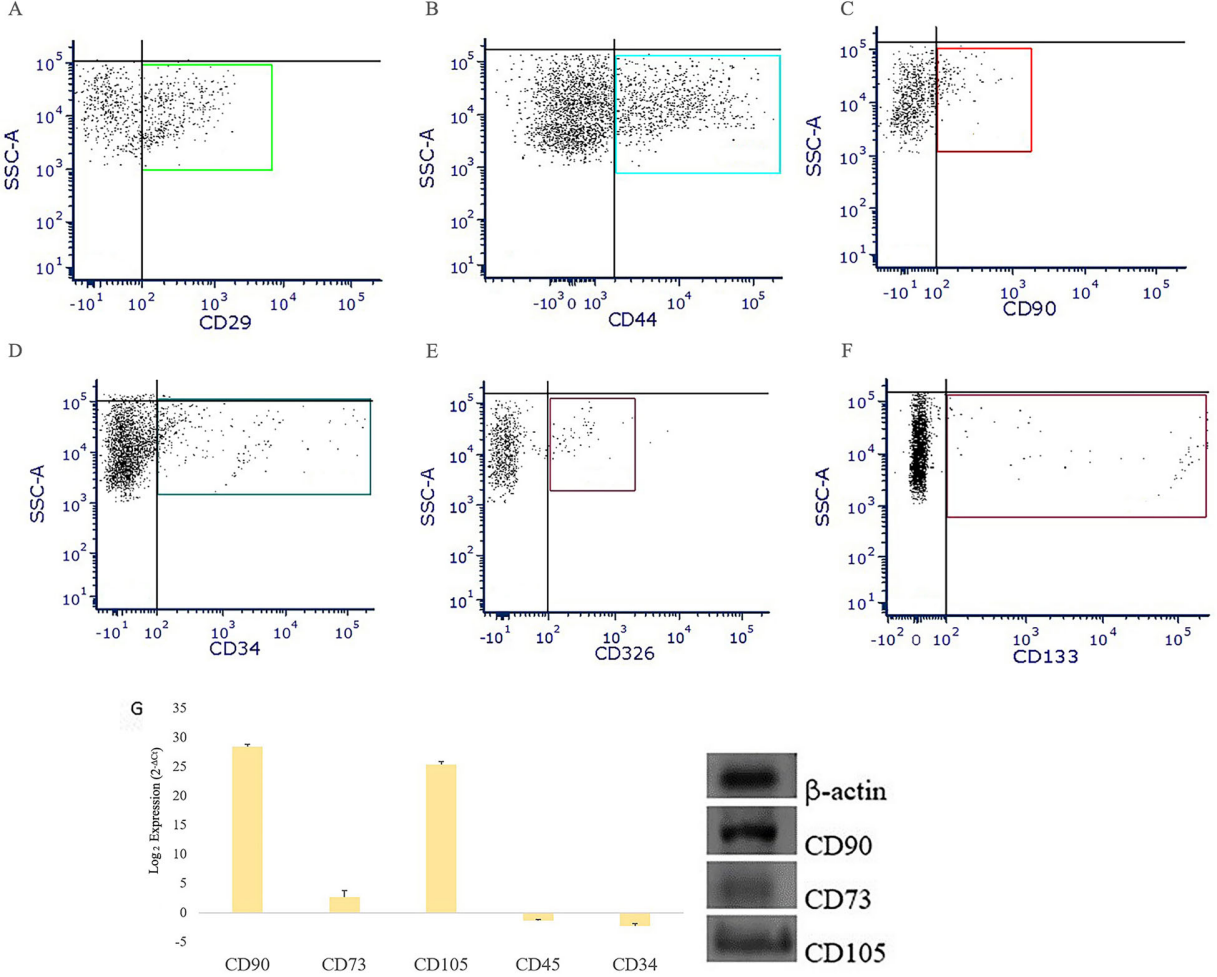
Flow cytometry to identify the epithelial cells, and mRNA analysis to verify the fibroblasts’ identity. **a-f** example dot plots. Flow cytometry scatter plots of the adherent epithelial cells side scatter (SSC)/ Fluorescence dot plots of each marker; epithelial cells were found positive for CD29 (**a**) and CD44 (**b**) lineages markers and also for CD90 (**c**), CD34 (**d**), CD326 (**e**), and CD133 (**f**) stemness markers; **g**) mRNA expression of genes involved in fibroblast characterization was determined by qPCR. Transcript levels were normalized to the *ACTB* reference gene using log_2_ (2^-ΔCt^) method. The data are presented as mean ± standard deviation (SD). The graph bar shows expression level of the genes *CD90*, *CD73*, *CD105*, *CD45*, and *CD34* of cultured fibroblasts. Gene expression was confirmed by 1.5% agarose gels

Epithelial stem cells in the skin are located within the hair follicle (HF) and below the bulge zone, in the supra-bulb bar region and within basal layer adjacent to the basement membrane [18–20]. These cells continuously-renew and possess the ability regenerate and repair tissue following damage. The presence of epithelial stem cells and proliferating epithelial cells among the epithelial cells isolated from the skin biopsies allowed for the development of a multi-layered in-vitro model of skin equivalent. In order to identify the presence of epithelial stem cells and committed epithelial progenitors on the primary epithelial cell culture, we evaluated the expression of well-established markers including the epithelial stem cell markers CD34 (CD34 molecule) [21] and the CD133 (Prominin-1) [22], CD90 [23] and the committed progenitor epithelial cell marker CD326 (epithelial cell adhesion molecule) [24]. Qualitative analysis of the epithelial cells in culture was performed for each biological replicate. Flow cytometry analysis was conducted for specific lineage markers CD29 and CD44. PE-IgG1 FITC-IgG1, PE-Cy5 and APC-IgG1 were used as negative controls to identify and quantify the percentage of positivity of the stained population for each marker. Results show that 45.8% ± 1.5 cultured epithelial cells were positive for CD29 (Fig. 3a example dot plot), 29.4% ± 2.2 positive for CD44 (Fig. 3c example dot plot) 12.6% ± 1.02 positive for CD90 (Fig. 3c example dot plot), and 7.67% ± 2.17 were positive for CD34 (Fig. 3d example dot plot), 5.97% ± 1.29 were positive for CD326 (Fig. 3e example dot plot) and 2.01% ± 0.51 were positive for CD133 (Fig. 3f example dot plot). Gene expression analysis of the primary fibroblasts culture showed a high expression of *CD105* (mean ± SD: 25.4 ± 0.07,) and *CD90* (28.5 ± 0.11), while a lower expression of *CD73* (2.68 ± 0.32SD) (Fig. 3g). Data also showed the absence of the expression of the genes encoding for hemopoietic markers *CD45* (− 2.62 ± 0.40) and *CD34* (− 3.69 ± 0.40). Analysis of the products on 1.5% agarose gel confirmed the presence of the qPCR products (Fig. 3g).

### Viability of the cell types used for the wound model

Cell viability tests were performed in triplicate on fibroblasts and epithelial cells. The viability (mean ± SD) of fibroblasts was 85.89 ± 0.51 and 85.67% ± 0.58% for epithelial cells.

### Effects of scratch injury assay and rTNF, on gene expression in skin equivalent model

**Fig. 4:**
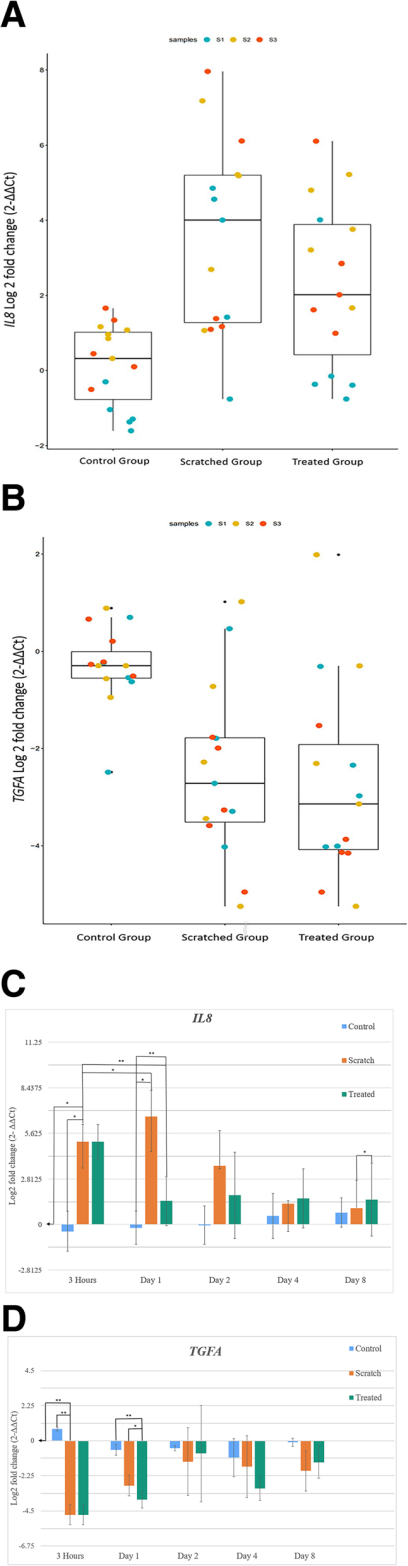
*IL8* and *TGFA* Gene Expression. Bar graphs showing the significant differences between the three conditions studied: control, scratched and treated, in relation to log fold change expression levels of the genes *IL8* (**a**) and *TGFA* (**b**). Bar graphs showing log fold change expression levels of the genes *IL8* (**c**) and *TGFA* (**d**) of the control, scratched and treated conditions for all time points at which they were measured. The mRNA expression was determined by qPCR. Relative transcript levels were normalized to the *ACTB* reference gene. The expression levels of the skin models at baseline time point, before scratching, were used as a calibrator. Data presented showing the log_2_ fold change. (2^−ΔΔCt^) method. The data are presented as mean ± standard deviation (SD). *P* values were worked out through a paired student t-test. *p* < 0.05 was considered as statistically significant and is shown in the figure where applicable (* *p* < 0.05, ** *p* < 0.01, *** *p* < 0.001)

We investigated the response of the skin equivalent cells in our experimental, leukocyte-depleted PRP-based skin equivalent model of a chronic wound associated with low skin perfusion. For this purpose, we performed real-time qPCR analysis to evaluate the level of gene expression involved in the inflammatory (*IL8)* (Fig. 4a) and proliferative *(TGFA)* (Fig. 4b) phases at various time points in an unscratched control condition and in response to the scratch injury and rTNF administration.

In these experiments, we monitored an unscratched control condition, an injured condition (scratched untreated), which received only the scratch and was used to verify the inflammation and proliferation of an untreated wound, and a treated condition which was administered a scratch injury and rTNF applied (Fig. 4a, b).

The expression level of IL8 was significatively different between the control condition and the scratched condition (*p* < 0.001), and also between the control condition and the treated condition (*p* < 0.01). No significant difference was found between the scratched condition and the treated condition. (Fig. 4a). The expression of IL8 increased significantly within 3 h of the scratch injury (*p* < 0.05) when compared with the control condition. Scratch injury led to the highest IL8 gene expression in the scratched condition at day 1 when compared with the control condition (*p* < 0.01). rTNF administration (30 ng/ml) led to a significant downregulation of IL8 in the treated group at day 1 when compared with the control condition (*p* < 0.001) (Fig. 4c).

The scratch assay induced IL8 upregulation at 3 h (fold change of 5.11) (*p* < 0.05) and this continued to increase up to day 1 in the scratched condition (fold change of 6.67) (*p* < 0.05). On the other hand, the administration of rTNF downregulated *IL8* expression (fold change of 1.45) (*p* < 0.01). This was followed by an upregulation (fold change of 1.81) on day 2. *IL8* expression decreased in the scratched condition after day 1 as the natural healing process took place and was lower than in the treated condition on day 8 (fold change of 1.54 and 1.01 respectively) (*p* < 0.05) (Fig. 4c). The expression of *IL8* decreased steadily in the scratched condition from day 2 onwards but increased slightly in the treated condition.

The expression level of TGFA was significatively different between the control condition and the scratched condition (*p* < 0.01), and also between the control condition and the treated condition (*p* < 0.001). No significant difference was found between the scratched condition and the treated condition (Fig. 4b).

The expression of TGFA decreased markedly within 3 h of the scratch injury(*p* < 0.01).

Both the scratched and the treated conditions showed a downregulation of TGFA within 3 h of the scratch when compared with the control condition (*p* < 0.01). rTNF administration (30 ng/ml) showed significant downregulation of TGFA at day 1 compared with the control condition (*p* < 0.01) and the treated condition (*p* < 0.05) (Fig. 4d)

### Protein secretion

We investigated the secretion of Interleukin 8 and transforming growth factor alpha in the cell culture medium of the three skin model conditions studied. These were measured at several time points (Table 1). Proteins levels were measured by ELISA assay.

**Table 1:** IL8 and TGFA concentration

Time points	Control	Scratched	Treated	Control	Scratched	Treated
	IL-8	IL-8	IL-8	TGFA	TGFA	TGFA
	pg/ml	pg/ml	pg/ml	pg/ml	pg/ml	pg/ml
Baseline	4.9 ± 1.7	4.7 ± 1.6	4.8 ± 1.7	11.3 ± 3	11.7 ± 2.5	14.6 ± 2
3 h	3.0 ± 0.6	39.1 ± 3.0	20.8 ± 1.6	7.3 ± 2.5	0.3 ± 0.5	0.1 ± 0.0
Day 1	3.6 ± 1.2	42.0 ± 3.2	25.1 ± 4.0	7.6 ± 1.9	0.7 ± 0.2	0.1 ± 0.0
Day 2	4.6 ± 1.9	42.1 ± 3.1	29.3 ± 0.5	2.0 ± 1.5	0.3 ± 0.4	0.1 ± 0.0
Day 4	4.4 ± 1.7	32.9 ± 4.0	23.5 ± 2.0	3.4 ± 1.4	5.6 ± 2.1	0.2 ± 0.3
Day 8	5.7 ± 3.4	50.8 ± 3.9	25.6 ± 3.4	1.2 ± 0.6	2.6 ± 1.3	0.1 ± 0.0

IL8 and TGFA were measured by Enzyme-Linked Immunosorbent Assay in the cell culture medium of the tree different conditions exanimated. The levels of IL8 and TGFA were equal in the three condition at the baseline time point. IL8 secretion remained stable in the control group for all time points while increased in the scratched and in the treated condition. TGFA secretion decreased in all time points

Results indicate equal quantities of IL-8 in all three conditions at the baseline time point before the scratch was induced (control: 4.9 pg/mL ± 1.7; scratched: 4.7 pg/mL ± 1.6; treated: 4.8 pg/mL ± 1.7). In the cell culture medium of the control condition, univariate amounts of IL8 for each time point were detected. In contrast, the scratched condition and the treated condition had higher IL8. There was a higher secretion or uptake of IL8 in the scratched condition compared with the rTNF treated condition at all the time points, suggesting that the administration of the rTNF reduced significantly the secretion of IL8.

Similar TGFA protein levels were identified in all three conditions at the baseline time point before scratching *(control*: 11.3 pg/mL ± 3; *scratched*: 11.3 pg/mL ± 2.5; *treated*: 11.7 pg/mL ± 2). The level of TGFA decreased with the time in the control condition. Low levels of TGFA were detected in the culture medium in both the scratched and the treated conditions. TGFA protein was absent in the treated condition, while proteins were detected in the scratched condition on day 2 and day 4.

## Discussion

**Fig. 5:**
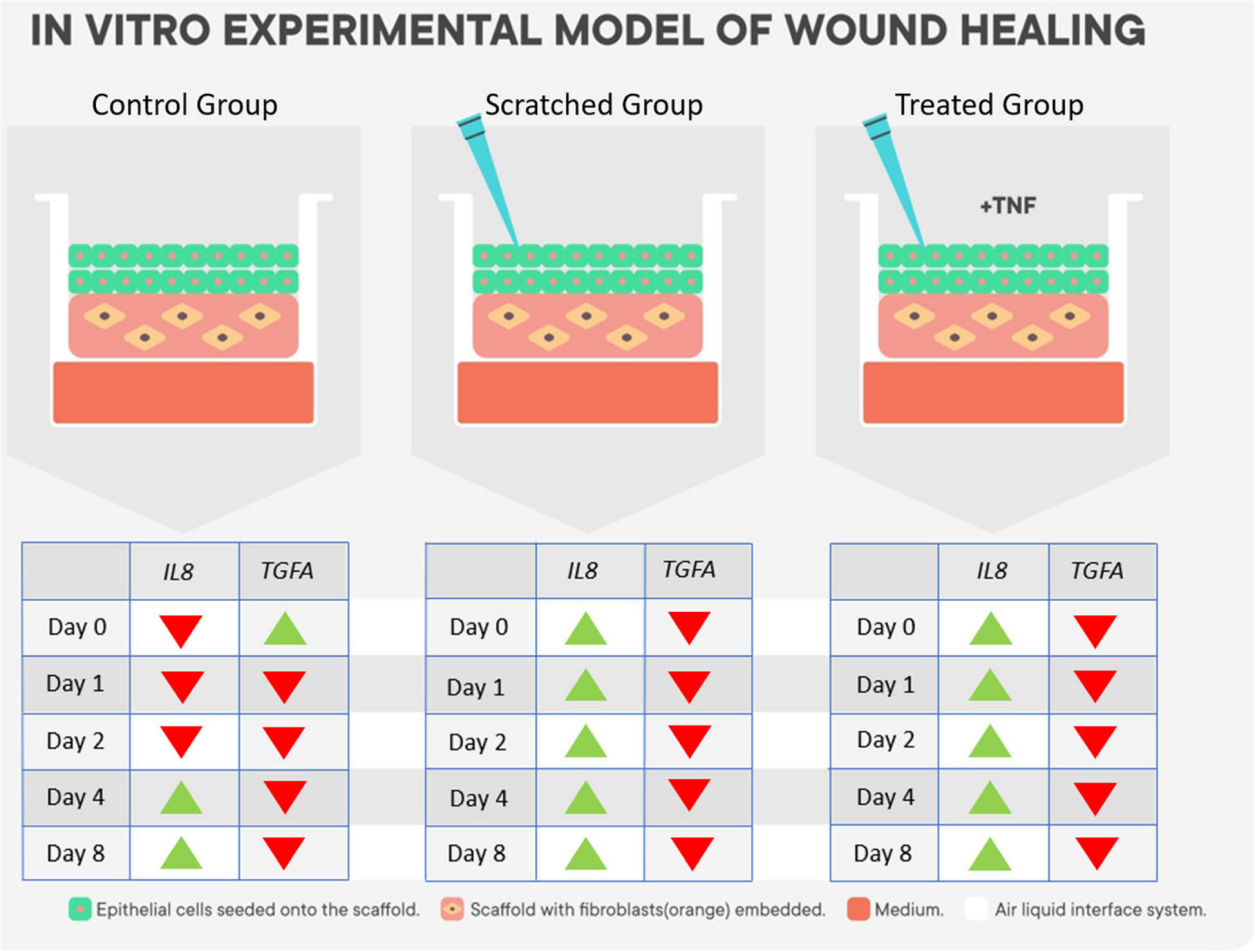
Experimental wound model and the changes in the expression of the genes. Schematic representation of the effects of scratch assay and rTNF administration, on gene expression in our skin equivalent model. *IL8* and *TGFA* expression levels were evaluated at different time points. Down arrows in red represent downregulation while up arrows in green represent upregulation

In this study we developed a novel skin wound model in order to observe the immune response during the wound healing process, by quantifying the gene expression of *IL8* and *TGFA* and measurable protein secretion. A control skin model consisting of a wounded condition without treatment was compared with a wounded (scratched) skin model with treatment. The treatment included the administration of 30 ng/mL rTNF which regulates genes that encode for inflammatory mediators [25]. This skin model is shown in Fig. 5.

Studies on monolayer cultures of keratinocytes integrated into culture media do not resemble the true nature of the physiological process of wound healing [26]. Multi-layered differentiated models are comparable to native skin and produce excellent results when analysing epithelial attachment, proliferation, differentiation, and dermal remodelling [27].

Our novel skin model consisted of a leukocyte-depleted, platelet-rich plasma scaffold, with embedded fibroblasts, as dermal equivalent and seeded keratinocytes as multi-layered epidermis. Calcium chloride was used as an activator to initiate the formation of autologous thrombin from prothrombin, forming a fibrin clot that provided a surface for keratinocyte seeding and enabled the skin cells to mature into stratum corner and basal, spinous and granular layers. The lack of leukocytes allowed the mimicking of typical chronic wounds of patients with poor skin perfusion and low leukocyte infiltration. We subsequently used our new wound model to analyze cytokine gene expression under two conditions: scratched, treated.

During the physiological wound process, platelets are early modulators of the healing process and the blood clot formed upon platelet activation provides a provisional “scaffold” containing fibrin molecule and plasma fibronectin. This occurs during the first 24 h after the injury and enables formation of a temporary matrix in the wound bed [28]. Therefore, our PRP-based scaffold as dermal equivalent resembles the physiological scaffolding formed during the haemostatic phase and required for the normal wound process.

The inflammatory response occurs within hours of damage at the affected area, and is a localized or systemic protective response. The response is activated by molecules expressed by pathogens or associated with tissue injury and are recognized by Toll-like receptors (TLRs) present on skin resident cells [29]. TLR activation in response to injury and inflammation is responsible for upregulation of *IL8* [30].

A significant upregulation of *IL8* expression was noted 3 h after the scratch injury when compared to the levels exhibited just before injury, thereby confirming the success of our scaffold in mimicking the wound response. On the other hand, the scratch injury exhibited a down regulatory effect on the expression of *TGFA*.

In addition to the induction of inflammation by chemokines, other molecules such as TNF promote the inflammatory response following wounding. It has been shown that the prolonged stimulation of TLR receptors causes downregulation of TLR2 and TLR4, which may act as a self-regulatory mechanism to prevent an overactive immune response [31] In our model we noted a significant downregulation of *IL8* following the administration of rTNF, which appears to indicate the delayed activation of the inflammatory response. An increase in *IL8* in the treated group occurred at a later time point compared with the scratched group.

TLR ligation triggers the innate immune responses mainly through the activation of macrophages and neutrophils, which release a large number of cytokines and growth factors stimulating the proliferation of fibroblasts and collagen biosynthesis [32]. It is well known that macrophages alter phenotypes from an M1 pro-inflammatory phenotype to an M2 pro-repair phenotype, leading to a reduction in inflammatory markers and a promotion of a proliferation phase, secreting PDGF, TGF-α, and bFGF [33].

In the clinical setting, the presence of a chronic venous insufficiency ulcer (CVIU) in a patient may result in an impaired and difficult wound healing process due to the persistence of a chronic inflammatory state, combined with a relative lack of angiogenesis. The absence of the progression from a chronic, pro-inflammatory state, with high levels of TNF-a, IL-8, RANTES, and MIP-1p, to an anti-inflammatory state, with increased cytokines released by recruiting mononuclear phagocytes, will delay the resolution of the inflammatory phase. A study conducted on patients with CVIU, specifically on tissue and fluid from the wound area, suggest that pro-inflammatory, anti-inflammatory, and macrophage-related cytokines may be important in the pathogenesis of such disease. Interestingly, in the untreated CVIU only IL-8 was present in levels higher than in normal skin [34]. This clinical observation could explain the high levels of IL8 in our wound model when compared with the control condition.

In a study by Moore et al., CVLUs show that macrophage activation is suppressed, leading to an impaired inflammatory response and reduced levels of cytokines and growth factors, which are fundamental for the recruitment of the resident skin cells [35]. Moreover, macrophages secrete PDGF, TGF-α, and bFGF, which modulate the epithelialization, collagen accumulation, and angiogenesis [36]. It was demonstrated that TGF-alpha secretion is regulated in response to the inflammatory cytokines [37, 38].

TGF-a mRNAs were isolated in both wound macrophages and epidermal keratinocytes at the wound edge [39]. Based on its gene expression level, TGFA can be considered as a biomarker of the early phase of re-epithelialization [40]. During the proliferative phase of wound healing, there is an increase in the migration and proliferation of fibroblasts and endothelial cells, as well as keratinocytes which secrete bFGF, EGF, VEGF, bFGF, and PDGF, TGF-α and KGF.

Conditions including CVLUs, venous hypertension, and pressure ulcers, are characterized by a reduced proliferation rate and a migration of resident fibroblasts, in comparison with patient-matched normal skin fibroblasts [41–44].

Cellular elements, growth factors and cytokines simultaneously play an important role in different phases of the healing process. Altering their production or any changes in their levels could account for the impaired healing observed in chronic wounds. In diabetic ulcers, the lack of new blood vessel formation results in poor afflux of inflammatory cells to release cytokines and growth factors [45].

As in the clinical setting of the CVLU, the absence and hence non activation of the macrophages could be explaining the deregulation of TGFA in our model. The results obtained with our model indicate that the scratch assay in both the untreated and treated conditions studied induced an inflammatory state, as shown by the lower gene expression of TGFA when compared with IL8. The absence of leukocytes, which promote the resolution of inflammation by releasing numerous potent cytokines, suggests a delay of the initiation of the proliferative phase. TGFA was downregulated when measured al all time points after scratching in both the scratched and treated conditions.

From this study we can suggest that the presence of a pro-inflammatory cytokine (TNF) regulate IL8 and TGFA production, by acting on the resident skin cells. The wound model indicated accurately how the resident skin cells induce IL8 and downregulate TGFA, in response to damage in the physiological and inflammatory state and in the absence of leucocytes afflux.

Further studies are required to investigate the functionality of our skin model scaffold in producing anti-inflammatory cytokines such as IL10, IL4, IFN-alpha, TGF-beta, following the administration of a scratch which mimics a skin wound to induce an inflammatory state. This will provide a better understanding of the skin resident cell response. Additionally, the functionality of our scaffold could be tested for new tissue growth under physiological and pathological wound conditions in view of its possible application in regenerative medicine.

## Conclusion

The leukocyte-depleted, platelet-rich plasma-based skin equivalent developed through this study could represent a useful in vitro model for further studies of typical chronic wounds related to poor skin perfusion and low leukocyte infiltration.

## Methods

### Isolation and cell culture of primary epithelial cells and fibroblasts

Sternal skin tissue biopsies discarded after surgical interventions were obtained from voluntary participants in this study with written consent form approved by the University of Malta Research Ethics Committee (UREC, ref. no 56120 L7). The voluntary participants did not demonstrate any chronic skin-related disease prior to the surgery. Biopsy tissue was required to create a skin equivalent model through a co-culture of fibroblasts and epithelial cells. The skin biopsies were washed in Dulbecco’s phosphate-buffer ed. saline (PBS) and then suspended in Dulbecco’s Modified Eagle Medium (DMEM) supplemented with antibiotics (50 μg/mL gentamycin, 100 units/mL penicillin, and 100 μg/mL streptomycin) as well as the antimycotic 0.25 μg/mL amphotericin B (Sigma-Aldrich, Milan, Italy). This was followed by overnight digestion at 4 °C with 1 mg/mL Collagenase/Dispase (COLLDISP-RO Roche, UK). The following day, they were incubated at 37 °C for 1 h with 0.25% trypsin-EDTA solution (Sigma-Aldrich) to separate the epidermis from the dermis.

The dermis and epidermis were cut into small sections of approximately 1 mm^2^ each and incubated separately at 37 °C for 2 h with 0.25% Trypsin-EDTA solution and mixing every 10 min. The enzymatic action was stopped by adding DMEM/F12 (Sigma-Aldrich) medium supplemented with 10% fetal bovine serum (FBS) (Thermo Fisher, Waltham, USA) and antibiotic (50 μg/mL gentamycin, 100 units/mL penicillin, and 100 μg/mL streptomycin) and antimycotic 0.25 μg/mL amphotericin B. The digested tissues were centrifuged at 100×g for 10 min at room temperature to obtain cell pellets. The epithelial cells derived from the epidermis were plated in a 6-well plate and cultured with Stemline Keratinocyte medium II supplemented with 5 μg/mL hydrocortisone and 10 ng/mL human recombinant keratinocyte growth factor (KGF) (Sigma-Aldrich). The dermal cells, fibroblasts, were plated in a 6-well plate and cultured with DMEM/F12 (1:1 mix) supplemented with 50 μg/mL L-ascorbic acid and 5 ng/mL human recombinant fibroblast growth factor (FGF-basic) (Sigma-Aldrich). Both complete culture media also contained 10% FBS, 100 μg/mL insulin, 50 μg/mL gentamycin, 0.25 μg/mL amphotericin B, 100 units/mL penicillin, and 100 μg/mL streptomycin (Sigma-Aldrich). Both cell types were cultured at 37 °C in a humidified atmosphere and 5% CO_2_.

### Flow Cytometry analysis of primary epithelial cells

Immunofluorimetric characterization of the epithelial cells was performed using monoclonal antibodies (moAbs) against Fluorescein Isothiocyanate (FITC) CD34 (Hematopoietic Progenitor Cell Antigen-1, Miltenyi Biotec, Surrey, UK), Phycoerythrin (PE) CD133/2 (Prominin-1, Miltenyi Biotec), PE-CD326 (epithelial cell adhesion molecule, clone 187 eBioscience, Waltham, USA) Alexa Fluor 488 CD29 (Integrin beta-1, clone TS2/16, Bio Legend, UK) Phycoerythrin-Cy5 (PE-Cy5) CD44 (CD 44 molecule, clone IM7, eBioscience) and Allophycocyanin (APC) CD90 (Thy-1, clone 5E10, Bio Legend) surface antigens. PE-IgG1 FITC-IgG1, PE-Cy5 and APC-IgG1 were used as corresponding isotype controls. Flow cytometry assay was done on adherent and 70% confluent epithelial cells. Cells were incubated at 37 °C for 10 min with 0.05% Trypsin-EDTA and the enzymatic action was stopped by adding 10% FBS in PBS. Cells were washed with PBS, and 200 μL of cell suspension was stained with 5 μL of each moAb for 20 min in the dark at room temperature. The cells were analysed with the FACS Aria II (FACS Diva version 6.1.2, Becton Dickinson, USA) and raw data analysis was performed using FCS Express 7, De Novo software. Initial forward scatter (FSC) and side scatter (SSC) distribution parameters of the cell populations were applied to exclude cell debris.

### Cell count and viability

The cell count and viability of the two cellular components keratinocytes and fibroblasts was assessed at the second passage in culture and before assembling the in vitro skin equivalent model. The assay was performed with Countess TM II Automated Cell Counter using a 0.4% Trypan Blue solution (Thermo Fisher Scientific) as per the manufacturer’s protocol. The analyses were performed in triplicate.

### Preparation of the leukocyte-depleted PRP-based skin equivalent

Pooled platelet products were provided by the National Blood Transfusion Centre. The platelet bags were collected as part of a voluntary blood donation from healthy donors. The products used for setting up the leukocyte-depleted PRP-based skin equivalent were 5 days old. The products were stored at constant agitation. The platelets were collected and stored in bags that allow oxygen and carbon dioxide exchange.

Pooled platelet concentrates were transferred into 50 mL centrifuge tubes and centrifuged for 20 min at 300 x g at 4 °C. The platelet pellet was resuspended in 5 mL residual plasma to form the PRP. One millilitre of PRP was mixed with 1 mL 20 mM calcium chloride (CaCl_2_) solution and 6 × 10^4^ cells/cm^2^ fibroblasts. The mixed solution was poured into a 24 mm trans-well with a 0.4 μm pore polyester membrane insert and a cell growth area of 4.67 cm^2^ (Corning Trans-well, Sigma-Aldrich) and left at 37 °C in a CO_2_ incubator for 24 h. The CaCl_2_ worked as activator for the formation of autologous thrombin and allowed the PRP-based derma equivalent to solidify.

The epithelial cells were seeded on the PRP-based derma equivalent with a density of 6 × 10^4^ cells/cm^2^ to establish the prototype skin equivalent. This skin model was cultured with an air-liquid interface system allowing the development of a multi-layered skin equivalent.

The medium culture was formulated as follows: DMEM/F12 medium and Stemline Keratinocytes medium in a proportion 1:1, supplemented with 10% FBS and antibiotic (50 μg/mL gentamycin, 100 units/mL penicillin, and 100 μg/mL streptomycin) and antimycotic 0.25 μg/mL amphotericin B. The culture system was provided with fresh medium every 3 days. Microscopical observation of the skin equivalent was performed every 3 days in order to observe the newly formed stratified epithelium. Once fully developed, after 4 weeks culture, the three biological skin equivalent replicates were collected and fixed for 24 h at room temperature in 10% neutral buffered formaldehyde for sectioning and processing using a short histology protocol, with an overnight incubation step in 60% ethanol. The tissue-equivalent sections were embedded in wax blocks, cut at 5 μm, and transferred to slides and stained with Mayer’s Haematoxylin-Eosin solutions (Sigma-Aldrich).

### The in vitro experimental model of wound healing

In order to create an in vitro experimental model of wound healing, a scratch injury of 1 cm length was inflicted using a 200 μL sterile tip. Administration of human rTNF (Bachem AG, Switzerland) was used to mimic an inflammatory condition. Three different conditions were evaluated in this study: *control*: unscratched condition, either a scratch or a rTNF were administered; *scratched:* a scratch injury was inflicted but no rTNF was subsequently administered; *treated*: a scratch injury was inflicted and 30 ng/ml of rTNF was administered 3 h after wound infliction to reproduce an inflammatory condition.

Skin equivalent models were collected at baseline (ie prior to wound infliction), at day 0 (3 h after wound infliction) and then at 1, 2, 4 and 8-day intervals after the wound was inflicted. Each condition was repeated in a biological triplicate.

### RNA extraction and qPCR of native fibroblasts and skin equivalent

RNA was extracted from 5 × 10^6^ adherent fibroblasts, and the leukocyte-depleted PRP-based skin equivalents using the Pure Link® RNA Mini Kit (Thermo Fisher Scientific). In brief, leukocyte-depleted PRP-based skin equivalents were harvested quickly and were immediately digested and homogenised with 45 μl of Digestion Buffer and 5 μl of Proteinase K using a homogenizer (Speed Mill PLUS Analytic Jena AG, Germany). The cycles of homogenisation were: 30 s high speed, 1 min pause, 30 s high speed. RNA was then extracted from the homogenised tissue according to the manufacturer’s protocol.

RNA quality control and measurement of the amount of total RNA were performed using a Nanodrop 2000 Instrument (Thermo Fisher Scientific). Complementary DNA (cDNA) was produced from 50 ng of RNA from each sample using the Revert Aid™ First Strand cDNA Synthesis Kit (Thermo Fisher Scientific). qPCR was performed using Rotor-Gene Q Series Software 2.1.0. (Qiagen, Valencia, California, USA). One microliter of the neat cDNA was amplified in a final volume of 20 μL with 5x HOT FIREPol Eva Green qPCR Super mix (Solis BioDyne, Tartu, Estonia) and primers for *IL8*, *TGFA*, *CD34*, *CD45*, *CD90*, *CD73*, *CD105*, and *ACTB* (Table 2). Thermal cycling proceeded for 12 min at 95 °C followed by 40 cycles at 95 °C for 15 s, 60 °C for 30 s, and 72 °C for 30 s. All real-time PCR reactions were performed in triplicate. The relative expressions of mRNAs were calculated using the comparative Ct method (2^^(−∆∆Ct^) and normalized against the endogenous reference gene *ACTB* (encoding β-Actin), and the data are reported as the mRNA fold change. The gene expression levels of the skin equivalent models at the baseline time point before scratching (non-injured state) were used as calibrators for all conditions evaluated.

**Table 2:** Primer sequences and amplicon sizes for each gene analysed in the study

Gene	Forward Primer (5′-3′)	Reverse Primer (5′-3′)	Fragment size
*CD34*	TGAAGCCTAGCCTGTCAC	ATAAGACCTCCAGCTGTGCG	180 bp
*CD45*	GTGTTTCATCAGTACAGACG	GCTGTCATTTCAACCACAAC	191 bp
*CD73*	ATGGTGTGGAAGGACTGATC	CATCGCTCAGAAAGTGAGG	310 bp
*CD90*	TGCTCTTTGGCACTGTGG	CTGCTCCTGCTCTCCCTCT	248 bp
*CD105*	GGGGTCAACACCACAGAG	CACATCCTGAGGGTCCTG	261 bp
*IL8*	GAGAGTGATTGAGAGTGGACCAC	AAACTGGGTGCAGAGGGTTGTG	90 bp
*TGFA*	GGTCCGAAAACACTGTGAGTGG	AAGAGCCCAGAGGAGGAGTTTG	108 bp
*ACTB*	AGTCCTAGCTACTCCGGAGGC	CGGCTATTCTCGCAGCTCAC	113 bp

### Enzyme-linked Immunosorbent assay

The secretion of Interleukin 8 and transforming growth factor alpha into cell culture media was determined using enzyme-linked immunosorbent assay (ELISA) (Thermo Fisher Scientific). The optical density (OD) at 450 nm of samples was read using a Mithras LB940 multimode microplate reader and the curve-fitting standard curve and data were analysed with Excel software. Culture medium that had no addition of cells was used as a blank for the subtractions of the sample measurements. Each biological replicate was run assessed in two technical replicates.

### Statistical analysis

R (version 3.6.3) was employed for the statistical analysis. Data are presented as mean ± standard deviation (SD). The difference between the means of the groups was assessed using the ANOVA test, followed by Tukey’s post hoc analysis to find the significantly different pairs. A paired student t-test was used to analyse the data of the three conditions at the different time points, with an alpha threshold of 0.05.

## Supplementary Information

**Additional file 1: Supplementary figure.** mRNA analysis to verify the fibroblasts identity. 1.5% Agarose gel showing the qPCR products of the qPCR performed on cultured fibroblasts. Lane 1: 100 bp ladder; lane 2: B-actin product; lane 3: CD73 product; lane 4: CD90 products; lane 5: CD105 product; lane 6: 1 kb ladder. Ladders used: 100 bp DNA Ladder Ready to Load Cat. No. 07–11-00050 and 1 kb DNA Ladder Ready to Load Cat. No. 07–12-00050, (Solis BioDyne, Tartu, Estonia).

## Data Availability

All data generated or analysed during this study are included in this published article or is available from the corresponding author on reasonable request.

## Abbreviations

APC: Allophycocyanin
bFGF: Basic Fibroblast Growth Factor
CaCl_2_: Calcium Chloride
CD: Cluster of differentiation
cDNA: Complementary DNA
Ct: Cycle threshold
DMEM: Dulbecco’s Modified Eagle’s Medium
EDTA: Ethylenediaminetetraacetic acid
EGF: Epidermal growth factor
FACS: Fluorescence-activated cell sorting
FBS: Fetal Bovine Serum
FITC: Fluorescein isothiocyanate.
FL: Fluorescence
FSC: Forward Scatter
FGF- basic: Fibroblast growth factor
KGF: Keratinocyte growth factor
MoAbs: Monoclonal Antibodies
mRNA: Messenger RNA
PBS: Phosphate-buffered saline
PDWHF: Platelet-Derived Wound Healing Factor
PDGF: Platelet Derived Growth Factor
PE: Phycoerythrin
PE-Cy5: Phycoerythrin-Cy5
PRP: Platelet Rich Plasma
qPCR: Quantitative PCR
RNA: Ribonucleic acid
rTNF: Recombinant TNF
SSC: Side Scatter
SD: Standard Deviation
T2DM: Type 2 Diabetes Mellitus
TGF-α: Transforming growth factor alpha
TLR: Toll-like receptors
TNF: Tumor Necrosis Factor
VEGF: Vascular endothelial growth factor
Δ Ct: Delta Ct
ΔΔ Ct: Delta Delta Ct
