# Supplementary material for: Novel leukocyte-depleted platelet-rich plasma-based skin equivalent as an in vitro model of chronic wounds: a preliminary study
Source: BMC Mol Cell Biol. 2021 May 10;22:28. doi: 10.1186/s12860-021-00366-6 (PMC8111747; doi:10.1186/s12860-021-00366-6)
Supplement: Supplementary file 1 — Additional file 1: Supplementary figure. mRNA analysis to verify the fibroblasts identity. 1.5% Agarose gel showing the qPCR products of the qPCR performed on cultured fibroblasts. Lane 1: 100 bp ladder; lane 2: B-actin product; lane 3: CD73 product; lane 4: CD90 products; lane 5: CD105 product; lane 6: 1 kb ladder. Ladders used: 100 bp DNA Ladder Ready to Load Cat. No. 07–11-00050 and 1 kb DNA Ladder Ready to Load Cat. No. 07–12-00050, (Solis BioDyne, Tartu, Estonia). [file 12860_2021_366_MOESM1_ESM.docx]

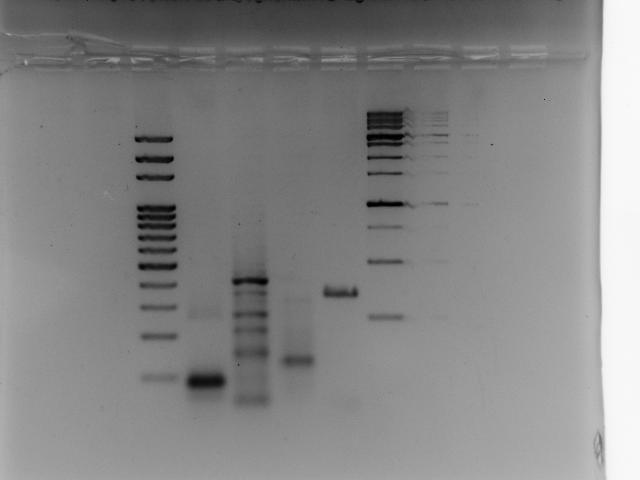


**Lane: 1 2 3 4 5 6**

**Supplementary figure: mRNA analysis to verify the fibroblasts identity.**

1.5% Agarose gel showing the qPCR products of the qPCR performed on cultured fibroblasts. Lane 1: 100bp ladder; lane 2: B-actin product; lane 3: CD73 product; lane 4: CD90 products; lane 5: CD105 product; lane 6: 1 kb ladder.

Ladders used: 100 bp DNA Ladder Ready to Load Cat. No.  07-11-00050
and 1 kb DNA Ladder Ready to Load Cat. No.  07-12-00050, (Solis BioDyne, Tartu, Estonia).
